# The fallacy of the equity-efficiency trade off: rethinking the efficient health system

**DOI:** 10.1186/1471-2458-12-S1-S3

**Published:** 2012-06-22

**Authors:** Daniel D Reidpath, Anna Elisabet Olafsdottir, Subhash Pokhrel, Pascale Allotey

**Affiliations:** 1 School of Medicine and Health Sciences, Monash University, Sunway Campus, Malaysia; 2 Centre for Public Health Research, Brunel University, UK; 3 Health Economics Research Group, Brunel University, UK

## Abstract

In the health systems literature one can see discussions about the trade off between the equity achievable by the system and its efficiency. Essentially it is argued that as greater health equity is achieved, so the level of efficiency will diminish. This argument is borrowed from economics literature on market efficiency. In the application of the economic argument to health, however, serious errors have been made, because it is quite reasonable to talk of both health equity being a desirable output of a health system, and the efficient production of that output. In this article we discuss notions of efficiency, and the equity-efficiency trade off, before considering the implications of this for health systems.

## Background

What is more important, a health system that delivers equitable health outcomes or an efficient health system? This meaningless question lies at the heart of the “equity-efficiency trade off.” It is semantically badly formed and the only response it should elicit is one of confusion or bemusement. Unfortunately, the debate about the equity-efficiency trade off (and its close and equally meaningless relative, the equality-efficiency trade off) elides the real question, and encourages knee-jerk rather than thoughtful consideration.

A more appropriate question would be, “what is more important for a population, a health system that delivers equitable (fairly distributed) health outcomes or a health system that maximises health gains?” The difference between the meaningless first question (which does not contrast outcomes) and the potentially meaningful second question (which does contrast outcomes) is critical. Unfortunately, the two questions are usually treated as equivalent. In fact, the literature is replete with examples of discussions about the equity-efficiency trade off. Sometimes the discussions make direct reference to a “trade off”, but often they rely on the juxtaposition of equity against efficiency with implicit reference to the trade off. The *World Health Report 2008*, is an example of the latter, implicit reference to the trade off, in which the goals of the healthcare system are simultaneously identified as ones of equity and of efficiency [1]. A more explicit trade off appears in the earlier World Health Report 2000 in which it is identified that “equity and efficiency can easily be in conflict” [2]. In the last two years, examples in the literature in which equity and efficiency are juxtaposed include papers on the trade off in the delivery of services and care such as HIV treatment [3], breast cancer services [4], and immunisation coverage [5]. Others discuss the trade off in more theoretical terms. One paper described the “iron triangle” in the health care system including equity and efficiency as two of the vertices [6], another reviewed the outcome dimensions of the primary health care system and identified efficiency of care and equity in health as separate outcome dimensions [7], and a third described efficient care as the bedrock of the health system “challenged” by the need for health equity [8].

The problem with trying to establish a trade off between a potential outcome, output, or goal of a health system such as health equity against efficiency, is that efficiency is not an outcome of a health system. Efficiency describes a functional relationship between inputs (such as money) and outputs (such as health gains) – but it is not in and of itself an outcome. The consequences of the semantic error have, we argue, misdirected efforts to develop efficient health systems. To bring clarity to the matter we revisit notions of efficiency, the equity-efficiency trade off, and the consequences of this for the efficient health systems.

## Discussion

Efficiency as an idea draws on the notion of *useful work* or *sought outputs*, with its origins in physics and engineering, and the transformation of heat energy into mechanical energy [9]. Not all heat energy becomes mechanical energy, and not all the mechanical energy produces useful work. Central to notion of a system's efficiency is the human valuation of the multiple outputs. Efficiency is thus a relationship between the inputs and the sought outputs.

In translating efficiency to economics, the same general idea applies. Given a certain level of inputs, the achieved level of sought outputs marks the efficiency of the system. The system that can produce the greatest useful work for a given level of inputs is the more efficient system. It is entirely a matter of judgement, however, which of the outputs are sought. For the owner of a factory the cost accounting might be quite straightforward, measuring efficiency in terms of widgets output per dollar input. In a large and highly complex system, producing myriad (often intangible) outputs, some of which become inputs for other parts of the system, determining efficiency can be difficult. Indeed, even on the factory floor the workers' views about the sought outputs (and therefore the systems efficiency) may not accord with the management's view. If one seeks more than a single output, then there is necessarily a trade off between the production of one output and the production of the other.

The idea that there is a trade off between achieving equity and achieving efficiency has its roots in economics, and the labour economist Arthur Okun's idea of the “big tradeoff” between equality and efficiency [10]. When Okun described the trade off, he used “efficiency” to refer narrowly to market efficiency, but it has since come to be used more generally to characterise any loss of efficiency in an economic system that occurs following increases in equality or equity [11]. In the health literature, efficiency is implicitly taken to mean the production of the greatest health gains for a given level of input (see for example, [12]). “Taken to mean” is used advisedly in the last sentence, because this is where the problem lies. Efficiency is not, in and of itself, useful work or a sought output. “Efficiency” describes a relationship between the useful work or a *sought output* of a system relative to the inputs. By treating efficiency as if it was one of the goals of a health system and equity was the other goal, the true nature of the trade off between health equity and health gain is hidden.

**Figure 1:**
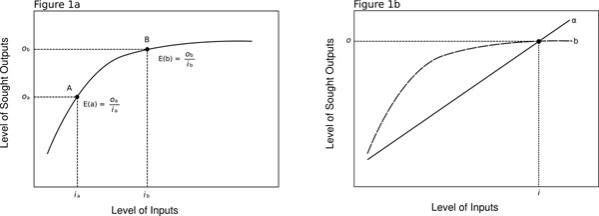
1a: Efficiency curve. 1b: Efficiency curve for interventions/policies α and β

More formally, consider the efficiency curve shown in Figure 1a. The x-axis represents arbitrary and increasing inputs, and the y-axis represents arbitrary and increasing outputs. By arbitrary, we mean that the inputs could be any number of scalable things including person-time, money, effort, or quantities of a specific good such as an antibiotic. The arbitrary outputs could, similarly be any number of scalable things including heart bypass procedures, life expectancy, decreasing levels of depression, or quantities of a specific good. Point **A** on the curve shows the level of output that can be achieved (*o*_a_) given a specific level of input (*i*_a_). The efficiency of A is the ratio of the level of output to the level of input: *E*(*a*) = *o_a_*/*i_a_*. It is possible to contrast the efficiency of two points on the same curve (**A** and **B**), finding, for instance (as in Figure 1a), that increasing the level of inputs from *i*_a_ to *i_b_* results in a greater level of outputs, from *o*_a_ to *o_b_*, but a reduced efficiency; i.e., *E*_a_>*E*_b_. A single efficiency curve could represent the relationship between the level of inputs and outputs for a given kind of intervention or policy.

Similarly one could contrast two different efficiency curves, representing a contrast of the efficiency of two different kinds of intervention or policy. Figure 1b shows the efficiency curves for interventions/policies α and β. Without calculation it is clear that for all levels of input less that *i*, the efficiency of β is greater than the efficiency of α (i.e., *E*_β_ >*E*_α_) because β produces greater levels of output than α for the same level of input, while for all levels of input greater than *i*, the obverse is true. At *i* level of input, the efficiency of α and β are the same (i.e., *E*_β_ =*E*_α_).

The efficiency curve for any intervention describes the trade off between inputs and outputs. Different levels of input achieve different levels of output, and given resource constraints, one can trade off inputs against outputs. Furthermore, the situation may well arise (as in Figure 1b) that as one changes the level of input, a less efficient policy or intervention becomes the more efficient one (e.g., α to β).

One can always compare two points on the same efficiency curve (i.e., for the same intervention), but it only makes sense to contrast two different efficiency curves (i.e., two different interventions) if they are scaled in the same way. That is, the metric for measuring the levels of inputs must be the same for both interventions, and the metric for measuring the levels of output must be the same for both interventions, and one could include social valuations of outcomes within the metric. The DALY for instance, which is commonly used as a health outcome measure reduces a multidimensional space of mortality, morbidity and explicit social valuations onto a common metric [13].

Now reconsider the idea of the equity-efficiency trade off in the context of the discussion so far. The efficiency curve describes the functional relationship between inputs and outputs. In the context of health systems, “efficiency” usually describes a relationship between some kind of input and a health outcome. This means that the so called, equity-efficiency trade off should be understood as a trade off between the level of input and the level of the health outcome, and the level of equity. To say that in fewer words, if no more meaningfully: the equity-efficiency trade off is a trade off between a trade off and equity. It's meaningless; and it appears that when the equity-efficiency trade off is described either explicitly or implicitly in the literature, the subtext is that the true sought outputs have not been fully included in the calculation of efficiency.

It is worth noting that there are various measures of efficiency used in the literature including technical efficiency, productive efficiency, and allocative efficiency [14]. The functional forms of these approaches conform to the example given above (Figure 1a). Other measures of efficiency could have different functional forms, but they inevitably refer to a functional relationship between inputs and outputs, and therefore succumb to the same problem of not being an outcome in and of themselves. The Pareto criterion, for example, requires that the sought outputs should not make anyone worse off. Applied to the maximisation of health gains, it means that the sought outputs maximise health gains, subject to the constraint that no individual's health is made worse off. For a given level of inputs, the Pareto efficient output is the one that maximises health gains subject to the Pareto criterion. Once the sought outputs are accepted, however, it makes no sense to trade off (Pareto) efficiency against some other output (such as some broader or different notion of equity) that is not sought.

Health systems can have various legitimate goals (outputs). It is theoretically possible that the goal of a health system is health equity alone, in which case one could (and should) seek to achieve that goal as with the greatest efficiency. To have a meaningful discussion about the efficiency of a health system, the real trade offs must be examined and this means that the goals of the health system must be identified. On a continuum of health gains and equity, possible goals of a health system include:

✯ Achieving the greatest health gains for a given input without regard to whether this means concentrating the gains in one (social) group: a traditional health outcomes focus,

✯ Achieving the fairest distribution of health for a given input without regard to the actual level of health achieved: a non-traditional outcome focus on (one form of) health equity, and

✯ Achieving an appropriate balance between the greatest health gains for a given input subject to the constraint of fairly distributing the health gains across social groups: an outcome balancing health equity and health gains.

This is not an idle point. By falsely identifying the trade off as one of efficiency against equity, health gains are implicitly elevated to the position of the “real goal” of a health system, and any other health system outcome is treated as an embarrassment that needs to be explained away. Given that health equity is debated at all, health gains are clearly not the only goal of interest. This is not a new point (e.g., [15]), but while we continue to view equity as oppositional to efficiency, the point has failed to achieve the prominence it requires [16]. Even those authors who recognise that the trade off is not between equity and efficiency in one context, fall into the trap of the equity-efficiency fallacy in another context [15].

The *World Health Report 2000* held that good health systems outcomes occurred on two separate dimensions: health gains and health equity [2]. This is not to say that the World Health Report was right, but it is clearly wrong to assume that health gains are the sole output of a health system without formally considering the trade off between health gains and other potentially worthy outputs of a health system such as health equity. It is impossible simultaneously to maximise two outcomes and if both gains and equity are indeed goals of the health system, then a function needs to be developed that combines the two outcomes and scales the combination according to a common metric (much as the DALY does for morbidity and mortality). It is this composite output that becomes the goal of the health system, and it is this output that should be pursued as efficiently as possible. A number of authors have proposed mechanisms for looking at the trade off between health gains and health equity, and the consequences of focusing on health gains to the exclusion of health equity (e.g., [17-19]). However, empirical, health systems research that has actually investigated preferences for the trade off, thereby identifying the actual balanced goal of a health system, is relatively unusual, and there is certainly no credible corpus of work informing this area.

Except in the vaguest terms of delivering health equity and delivering health gains, we do not really know what the final goals of a health system ought to be; and in all likelihood it will be different in different places. It is likely for instance that the view about the appropriate trade off between health gain and health equity will vary from setting to setting – further complicated by the fact that “health equity” has myriad meanings as does “health gain”. Nonetheless, what is very clear, is that in order to create efficient health systems, we need to cease discussing the meaningless trade off between equity and efficiency, and develop clear goals for an efficient health system that balances health equity and health gains – two desirable outputs of a health system.

As a final note, in the discussion we have tended to focus on goals that that might be described as final health outcomes. The argument, however, generalises to goals that might be described as intermediate health outcomes such as access to services, waiting time, etc., and by extension the equity related problems of the intermediate outcomes, such as equity of access.

## Summary

The notion of an equity-efficiency trade off has misdirected thinking. Efficiency is about the functional relationship between *useful work* or *sought outputs* of a system or process and the level of inputs. By juxtaposing equity and efficiency in the way it is traditionally done in the literature, we presuppose that the real sought output of the system is (a) known and agreed, and (b) not equity. This leads to a paradox, because either we will fail to achieve the greatest quantum of the outputs that we really seek by diverting attention to unsought outputs in the domain of equity, or equity is a part of the outputs that we really seek, in which case there is no trade off. By focusing attention on the true trade off, between the different sought outputs (such as health gains and health equity) then a rational discussion can develop around the appropriate balance between the sought outputs of a health system, and the most efficient way of achieving those outputs.

## Competing interests

The authors declare that they have no competing interests.
